# Transfer RNA-derived small RNA: an emerging small non-coding RNA with key roles in cancer

**DOI:** 10.1186/s40164-022-00290-1

**Published:** 2022-06-03

**Authors:** Xinliang Gu, Yu Zhang, Xinyue Qin, Shuo Ma, Yuejiao Huang, Shaoqing Ju

**Affiliations:** 1 Medical School of Nantong University, Nantong University, Nantong, China; 2 Department of Laboratory Medicine, Affiliated Hospital of Nantong University, Xisi Road, No. 20, Nantong, China; 3 Research Center of Clinical Medicine, Affiliated Hospital of Nantong University, Nantong, China; 4 Department of Medical Oncology, Affiliated Hospital of Nantong University, Xisi Road, No. 20, Nantong, China

**Keywords:** tRNAs, tsRNAs, Biological function, Cancer, Biomarker

## Abstract

Transfer RNAs (tRNAs) promote protein translation by binding to the corresponding amino acids and transporting them to the ribosome, which is essential in protein translation. tRNA-derived small RNAs (tsRNAs) are derived fragments of tRNAs that are cleaved explicitly under certain conditions. An increasing amount of research has demonstrated that tsRNAs have biological functions rather than just being degradation products. tsRNAs can exert functions such as regulating gene expression to influence cancer progression. Their dysregulation is closely associated with various cancers and can serve as diagnostic and prognostic biomarkers for cancer. This review summarizes the generation, classification, and biological functions of tsRNAs, and highlights the roles of tsRNAs in different cancers and their applications as tumor markers.

## Background

Transfer RNAs (tRNAs) are members of the small non-coding RNA (sncRNA) family and play an essential role in protein translation [1]. RNA polymerase III (Pol III) can transcribe tRNA genes into precursor tRNAs (pre-tRNAs), which then undergo a series of modifications and treatments to become mature tRNAs eventually [2, 3]. Mature tRNA possesses a conserved cloverleaf-type secondary structure consisting of a D-loop, anticodon loop, T-loop, variable loop, and an L-type tertiary structure maintained by hydrogen bonds [4]. In the process of protein translation in organisms, tRNAs mainly recognize codons on mRNAs through their anticodon loop structure and transport them to ribosomes by binding the corresponding amino acids through the principle of base complementary pairing to facilitate protein translation [5, 6].

tRNA-derived small RNAs (tsRNAs) were first discovered in the urine of cancer patients in the late 1970s. At that time, tsRNAs were considered random degradation products and did not attract widespread attention [7–9]. However, with the rapid development of high-throughput sequencing technology, more and more sncRNAs are being gradually identified. In recent years, many experiments and studies have proved that tsRNAs are derived fragments of pre-tRNAs or mature tRNAs produced by specific cleavage under specific environments, which have biological functions rather than just degradation products [7, 10].

In this review, we will describe the generation, classification, and biological functions of tsRNAs, emphasizing the role of tsRNAs in different cancers and their clinical application value as tumor markers.

## The generation and classification of tsRNAs

**Fig. 1:**
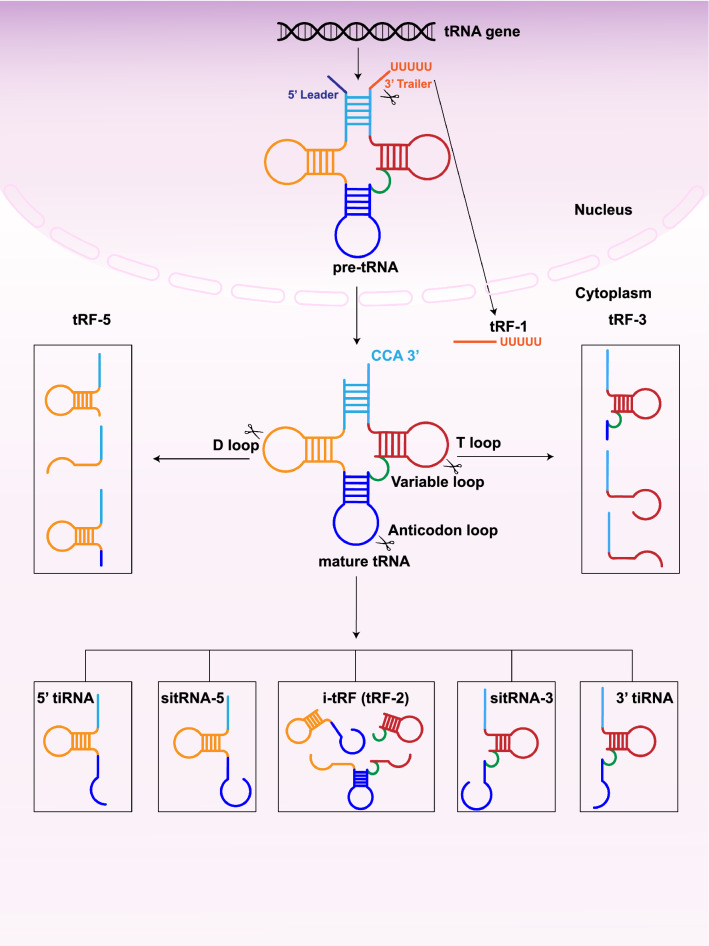
Biogenesis and classification of tsRNAs. tsRNAs can be divided into two species. The first type is tRFs, which can be divided into four subclasses, tRF-1, tRF-3, tRF-5, and i-tRF (tRF-2), based on the position of cleavage. The second type is tiRNAs, classified into 3′ tiRNA and 5′ tiRNA

Depending on the cleavage location on the tRNAs, tsRNAs can be divided into different species. The first type is tRNA-derived fragments (tRFs), which are about 14–30 nt in length and originate from pre-tRNAs or mature tRNAs [11, 12]. The second type is tRNA halves (tiRNAs), which are about 31–40 nt in length and are mainly produced by Angiopoietin (ANG), cleaving the anticodon loop of mature tRNAs under a variety of stress conditions [13]. Among tRFs and tiRNAs, many different classifications have been derived depending on the specific cleavage site (Fig. 1). In addition, sex hormone-dependent tRNA-derived RNAs (SHOT-RNAs) are produced under sex hormone-induced conditions [14].

tRFs are close in length to microRNAs (miRNAs) and can be divided into four subclasses, tRF-1, tRF-3, tRF-5, and i-tRF, based on the positions of cleavage of ANG, Dicer, or other RNases on mature tRNAs or pre-tRNAs (Fig. 1). tRF-1, also called 3′ U-tRF, is derived from the 3′ end of pre-tRNA, digested by ribonuclease Z, and has a characteristic poly-U sequence [15]. tRF-3, also known as 3′ tRF, is produced by nucleic acid endonuclease cleavage on the T-loop of mature tRNAs and contains a specific CCA structure at the 3′ end of the mature tRNA [16, 17]. tRF-5, also referred to as 5′ tRF, is derived from the 5′ end of mature tRNA and is a tRNA-derived fragment produced by Dicer cleavage in the D-loop or the region between the D-loop and the anticodon loop of mature tRNA [18]. The last type is i-tRF or tRF-2, which is cleaved from the internal structure of mature tRNA and does not contain the 5′ end or 3′ end of mature tRNA. Some types of it were found to be produced in breast cancer (BC) cell lines under hypoxic conditions, but the exact mechanism of its production remains unclear [19–21].

tiRNAs are tRNA half-molecules produced by ANG-specific cleavage at the anticodon loop of mature tRNA under various stress conditions such as hypoxia, UV radiation, heat shock, oxidative stress, viral infection, and phosphate deficiency [22]. tiRNAs can be classified into two types, 3′ tiRNA and 5′ tiRNA [23, 24] (Fig. 1). 3′ tiRNA starts at the 3′ end of the mature tRNA and ends at the anticodon loop cleavage site, whereas 5′ tiRNA extends from the 5′ end of the mature tRNAs anticodon loop [25, 26]. If the generated fragment is longer than the tiRNA, the fragment will be called stress-induced tRNA-3 (sitRNA-3) and stress-induced tRNA-5 (sitRNA-5) [27].

## Biological functions of tsRNAs

tsRNAs have multiple biological functions, including regulation of gene expression, regulation of translation, and regulation of epigenetics, exerting their biological roles in various ways.

### Regulation of gene expression

**Fig. 2:**
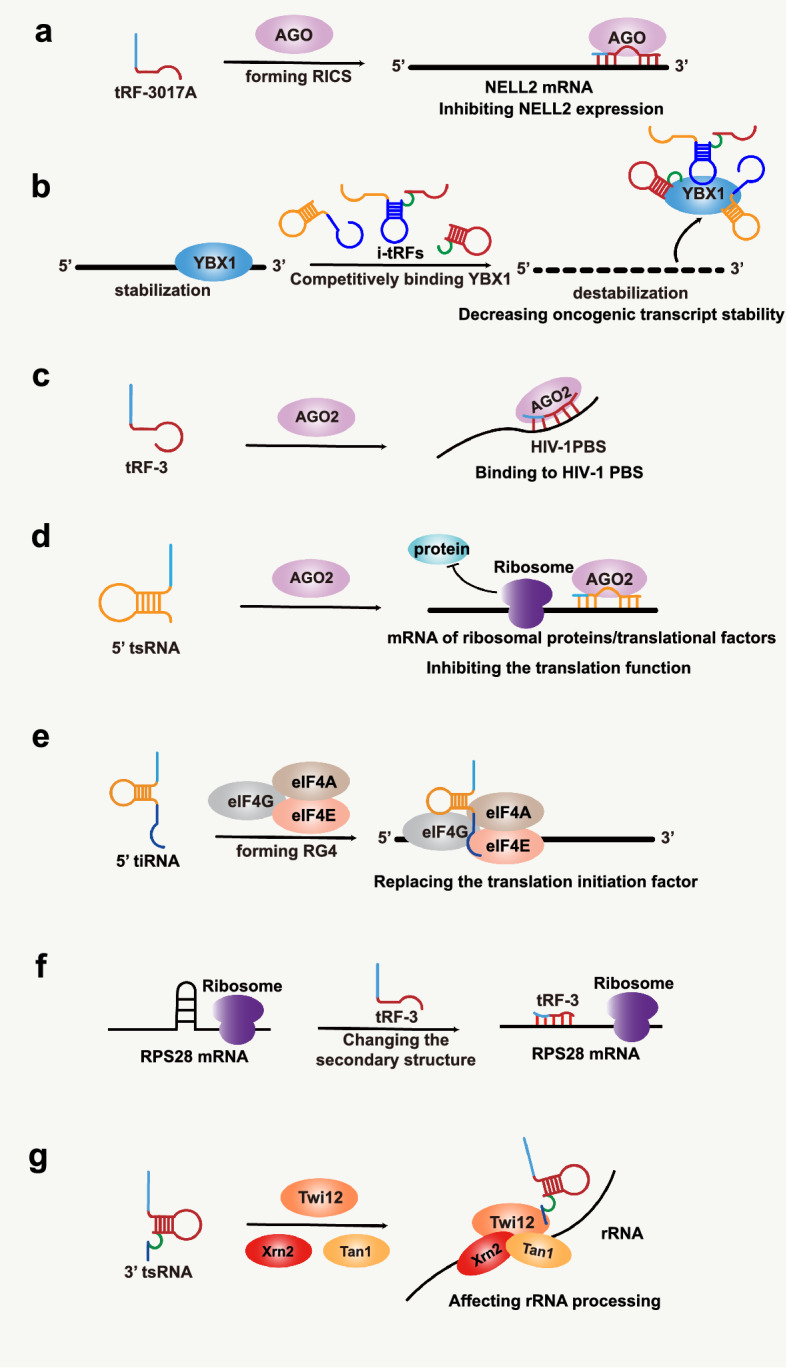
Biological functions of tsRNAs. **a** tRF-3017A forms RICS with AGO to inhibit NELL2 expression. **b** i-tRFs decrease oncogenic transcript stability through competitive binding of YBX1. **c** tRF-3 inhibits HIV gene expression by binding to HIV-1 PBS with AGO2. **d** 5′ tsRNA inhibits the translation function of mRNAs of ribosomal proteins and translational factors through AGO2, which in turn affects global translation. **e** 5′ tiRNA assembles with eIF4E/G/A to form RG4 for translational inhibition. **f** tRF-3 enhances RPS28 mRNA translation by altering the secondary structure of mRNA. **g** 3′ tsRNA binding to Twi12 and Xrn2, Tan1 affects rRNA processing

tsRNAs can regulate gene expression in multiple ways. Similar to the regulatory mechanism of miRNAs, tsRNAs can participate in the formation of RNA-induced silencing complex (RISC) and regulate the stability of mRNA through RISC binding to the 3′ untranslated region (3′-UTR) of target genes via the post-transcriptional pathway to regulate gene expression [28]. In gastric cancer (GC), tRF-3017A can bind to Argonautes (AGO) protein to form RISC and downregulate the expression of the tumor suppressor gene neural EGFL like 2 (NELL2), thus affecting the migration, invasion, and other functions of GC cells [29] (Fig. 2a). Besides, tRF5-GluCTC is important for RSV replication and host gene regulation at the post-transcriptional level. RNA pull-down experiments revealed that tRF5-GluCTC could exert regulatory effects through binding to AGO4 protein, while AGO1 directed tRF5-GluCTC to perform gene regulation in an atypical manner that remains unclear [30]. In addition, tRF-T11 derived from Chinese redbud can target the 3′-UTR of oncogene TRPA1 mRNA by interacting with AGO2 to suppress TRPA1. This research holds great promise for the development of novel RNA drugs derived from nature [31].

tsRNAs can also act as protein decoys that influence the stability of the targeted RNAs and regulate gene expression by competitively binding RNA-binding protein (RBP) [32]. Under hypoxic conditions, BC cell lines produce a series of tRFs that reduce the stability of multiple oncogenic transcripts by competitively binding the 3′-UTRs of the RBP Y-box binding protein 1 (YBX1), which decreased the expression of oncogenes in the post-transcriptional pathway, leading to the suppression of tumor growth [20] (Fig. 2b). In addition, 5′ tsRNA can reduce the stability of oncogenic transcripts c-Myc by competitively binding insulin-like growth factor 2 mRNA binding protein 1 (IGF2BP1), which inhibits the expression of c-Myc mRNA and thus promotes the differentiation of stem cells in mouse embryonic stem cells (mESCs) [33]. The above two studies add a new research direction to the way tsRNAs regulate gene expression.

Furthermore, it has been reported that tRF-3 derived from tRNA^Lys^ can inhibit human immunodeficiency virus-1 (HIV-1) reverse transcription by binding to AGO2 and thus antisense complementation with the HIV-1 primer-binding site (PBS), which inhibits HIV viral gene expression [34] (Fig. 2c). In host cells, tRF-3019 can be used as a primer to initiate and promote reverse transcription of respiratory syncytial virus (RSV), which in turn accelerates the self-replication of RSV viral genes and increases the infection efficiency and reproduction of RSV. Therefore, controlling the level of tsRNAs may become a new approach to controlling viral infection [35–37].

### Regulation of translation

tsRNAs can regulate the translation process of biological proteins in different ways, specifically in two different ways, AGO-dependent and AGO-independent ways [38]. Among them, the AGO-dependent translational repression approach in which tsRNAs can preferentially bind to AGO and subsequently target conserved sites in mRNAs through the 7-mer motifs of tsRNAs for antisense matching, ultimately reducing the translational activity of mRNAs, but did not affect the mRNA levels of tsRNAs target genes [38, 39]. In Drosophila, the regulation mediated by tsRNAs is dependent on AGO2. 5′-tsRNA can inhibit the translation of mRNAs of ribosomal proteins and other translational factors through antisense pairing, thus inhibiting overall protein synthesis [38, 40] (Fig. 2d). A previous study found that in human HEK293 cells, tRFs are associated with 1, 3, and 4 of the AGO family and may silence mRNA translation by binding to the AGO protein family [16]. Maute et al. found that a tRF named CU1276 is downregulated in B-cell lymphoma with a DICER1-dependent biogenesis property similar to miRNAs and could physically associate with AGO proteins, which can modulate proliferation and the DNA damage by inhibiting the translation of RPA1 [41]. Furthermore, in rhizobia, three tRFs were shown to exert translational repression by hijacking AGO1 to regulate host genes associated with rhizome initiation and development [42].

In AGO-independent translation regulation, tsRNAs can regulate translation by assembling to form RNA G-quadruplexes (RG4), affecting ribosome production, function, and structure [39, 43, 44]. Endogenous 5′-tiRNA molecules produced by stress can assemble to form RG4 structures that replace the translation initiation factor eIF4E/G/A on the mRNA cap and exert a translational repressive effect on mRNA [38, 45, 46] (Fig. 2e). Ribosomes are known to be ribonucleoprotein machines that decode the genetic information of mRNAs into polypeptides. Non-coding RNAs play an essential role from the transcription and processing of precursor rRNAs to the assembly of ribosomes [47]. Kim et al. showed that LeuCAG3’ tsRNA could promote the protein translation process of mRNAs through base complementary pairing binding to the mRNA of ribosomal protein S28 (RPS28), which is related to ribosome production, resulting in a change in the secondary structure of the RPS28 mRNA and thus affecting ribosome production [43] (Fig. 2f). Subsequently, further studies revealed that the regulatory effect of LeuCAG3’ tsRNA on the translational process of RPS28 in humans and mice was after the translation initiation phase. The tsRNA enhanced and promoted the translation of RPS28 mRNA by binding to its coding sequence (CDS) and non-coding 3′-UTR sequence to alter its secondary structure. Inhibition of LeuCAG3′ tsRNA expression significantly inhibited the translation process and decreased the expression of RPS28 protein [44]. It has been found that tRF-3 is associated with the PIWI protein Twi12 protein, which is essential for cell growth. The complex formed by them can affect rRNA processing and thus regulate translation by binding to Xrn2 and Tan1 [48] (Fig. 2g). In addition, under stress conditions, the salt-loving archaeon H. volcanii can produce Val-tRFs, which function as competitive binders with mRNA and can inhibit peptide bond formation and thus protein translation by binding to the small ribosomal subunits immediately adjacent to the mRNA channel [39]. In contrast, in the unicellular protozoan Trypanosoma brucei, 3′ tsRNA^Thr^ produced in a stressful environment can integrate into the ribosome and enhance mRNA loading and thus have an effect on global translation [49].

### Regulation of epigenetics

tsRNAs also have functions in regulating biological epigenetics. tsRNAs, similar to PIWI-interacting RNAs (piRNAs), can also bind to PIWI proteins to form complexes that play important roles by regulating genes associated with germ cell and stem cell developmental pathways [50–52]. Previous studies in hfd-fed mice found that sperm tiRNAs contribute to the transmission of acquired metabolic disorders to the next generation, identifying a role for tsRNAs in intergenerational inheritance as well as in the transmission of metabolic phenotypes [53, 54]. In mice, it has been found that tsRNAs are enriched during sperm maturation and early embryonic development and may play a relevant role by replacing piRNAs in cells or tissues where they are not available [55]. In contrast, the conversion of RNA load from piRNAs to tsRNAs during sperm maturation also suggests that tsRNAs are involved in epigenetic regulation [56]. A related study showed that mice deficient in DNA methyltransferase-2 (DNMT2) prevented high-fat diet-induced intergenerational genetic disorders [57], while the absence of DNMT2 abolished alterations in sperm tsRNAs modification and expression, suggesting a key role for tsRNAs in epigenetic mechanisms [58]. The above studies indicate that tsRNAs are equivalent to new piRNAs in playing significant roles in the epigenetic regulation of organisms. Furthermore, tsRNAs are capable of regulating histone methylation in human monocytes, and there may be additional tsRNAs that may be involved in the epigenetic regulation of somatic cells that are temporarily undiscovered and unstudied [59]. Besides, tRFGluTTC could reduce the expression of Cyclin D1, Cyclin-dependent kinase 4, and Cyclin E, which are factors essential for the maintenance of the G1/S phase in mammalian cells, to promote the proliferation of preadipocytes and reduce the expression of fatty acid synthesis-related genes to affect lipid accumulation, playing an essential role in adipogenesis [60–62].

## Dysregulation of tsRNAs in cancer and related roles

With the increasing development of high-throughput sequencing technology, a growing number of studies have found that dysregulation of tsRNAs is closely associated with the development of multiple cancers. Currently, tsRNAs have been reported to be dysregulated in cancers such as BC, colorectal cancer (CRC), GC, hematological tumors, etc. A wide range of tsRNAs has been found to possess the ability to regulate the proliferation and metastasis of tumor cells and influence the tumor cell cycle and apoptosis. Next, we will discuss the role of tsRNAs in different cancers.

### BC

BC has surpassed lung cancer (LC) as the fifth cause of global cancer death and the leading cause of cancer incidence worldwide [63]. Many kinds of different non-coding RNAs have been found to influence the development of BC, among which tsRNAs can regulate BC progression in several ways. Goodarzi et al. found that a series of novel tRFs produced by BC cell lines under hypoxic conditions could bind to YBX1, decreasing the stability of YBX1 endogenous oncogenic transcripts and inhibiting the progression of BC. They speculated that the interaction between tRFs and YBX1 may be only a small part of the general regulatory network of sncRNAs and RBPs [20]. Another study found specific interactions between tRF3E and nucleolin (NCL) by RNA Pulldown assays. In BC, the diminished competitive binding of tRF3E to NCL enhanced the translational repression of p53 mRNA by NCL, leading to decreased expression of the cancer suppressor gene p53 and promoting the progression of BC [64]. In addition to the RBP mechanism, tsRNAs can also play a regulatory role by binding to the target genes. 5′-tiRNA^Val^ was significantly decreased in BC tissues and cell lines and can directly bind to frizzled class receptor 3 (FZD3) to inhibit BC progression by suppressing the FZD3/Wnt/β-Catenin signaling pathway, acting as an inhibitory factor in BC [65]. Besides, tRF-19-W4PU732S, which is significantly highly expressed in BC, could promote the malignant progression of BC by inhibiting ribosomal protein L27a (RPL27A) [66]. While ts-112 and RUNX family transcription factor 1 (RUNX1) were negatively correlated in BC cells versus normal breast epithelial cells, RUNX1 may exert its cancer-suppressive function by inhibiting ts-112 to prevent over-proliferation of normal breast epithelial cells [67]. tsRNAs can also promote immunotherapy in BC. Shan et al. evaluated the impact of the interaction between tRFs and T cell activation on the survival of BC patients by Kaplan–Meier survival and multivariate Cox regression models and identified five tRFs-related pathways associated with survival by Spearman analysis, providing new BC treatment targets to improve immunotherapy in BC [68]. In triple-negative BC (TNBC), dysregulation of tsRNAs caused disorders of various mRNAs, which consequently had an impact on immune response pathways, cell signaling, chemoresistance, and energy metabolism [69]. Under hypoxic conditions, two tsRNAs (tDR-0009 and tDR-7336) involved in maintaining cellular responses to interleukins were significantly upregulated in the SUM-1315 cell line, which may be a potential mechanism by which hypoxic conditions induce the generation of tsRNAs to promote adriamycin resistance in TNBC, suggesting that specific tsRNAs may be a regulatory molecule involved in chemotherapy resistance in hypoxic TNBC and have the potential to become a new biomarker and intervention target in TNBC too [70]. In addition, the expression of tRFLys-CTT-010 is increased in TNBC and interacts with the glucose-6-phosphatase catalytic (G6PC) subunit to regulate cellular lactate production and glycogen consumption levels, which promotes the proliferation of cancer cells, suggesting that tRFLys-CTT-010/G6PC and the glucose metabolism regulatory axis may be a new therapeutic target for TNBC [71]. In summary, in BC, tsRNAs can regulate the malignant progression of BC through mechanisms such as RBP and target genes and can also regulate BC progression in multiple ways by affecting immunotherapy, chemoresistance, and energy metabolism, providing new markers and targets for the diagnosis and treatment of BC in the clinical practice.

### CRC

CRC is a clinically common cancer with the third highest incidence rate and the second-highest mortality rate [63]. In CRC, tsRNAs have also been found to have an essential role in regulating the progression of CRC by modulating multiple signaling pathways. The expression of tsRNAs was susceptible to hypoxic conditions, where 5′ tiRNA-His-GTG was regulated by the hypoxia/HIF1α/Ang axis, with increased expression under hypoxia, and could directly target LATS2 to inhibit the Hippo signaling pathway and promote the expression of proliferation and anti-apoptosis related genes, thus promoting CRC development both in vivo and in vitro [72]. Also under hypoxic conditions, Luan et al. found that Dicer1 could induce increased expression of tRF-20-MEJB5Y13, which leads to enhanced migration and invasion of CRC cells, providing new insight into the studies regarding hypoxia signaling regulatory tsRNAs in CRC [73]. Huang et al. identified a fragment that could be derived from both tRNA^Leu^ and pre-miRNA, which they named tRF/miR-1280. The expression of tRF/miR-1280 was decreased in CRC, and mechanistic studies revealed that tRF/miR-1280 could bind to Notch ligand JAG2 leading to inactivation of Notch signaling, which ultimately inhibited CRC growth and metastasis. This study demonstrated the possibility that miRNAs are derived from tRNAs and provided a new insight for investigating tsRNAs [74]. Besides, tsRNAs could also regulate the metastatic ability of CRC. The expression of ANG was elevated in CRC tissues, promoting CRC growth and metastasis both in vivo and in vitro. Meanwhile, a series of tiRNAs produced by ANG cleavage are enriched in both CRC tissues and highly metastatic cell lines and are involved in the process of ANG-promoted CRC metastasis, suggesting that the ANG/tiRNAs/cell migration invasion regulatory axis may be a new therapeutic target for patients with metastatic CRC [24]. Besides, the expression of tRF-phe-GAA-031 and tRF-VAL-TCA-002 was significantly upregulated in CRC tissues, suggesting that they may play an essential role in the growth and metastasis of CRC and are potential biomarkers as well as therapeutic targets for CRC [75]. In addition, Wang et al. established the expression profiles of tsRNAs in CRC by high-throughput sequencing. They analyzed and predicted the targets of tsRNAs, the relationship between tsRNAs and mRNAs, and constructed gene regulatory modules for tRFs and tiRNAs simultaneously, which provided directions for subsequent studies on the functions and mechanisms of tsRNAs in CRC [76]. In conclusion, in CRC, tsRNAs could affect the progression of CRC by regulating multiple signaling pathways and play an essential role in the metastasis of CRC, providing new therapeutic targets for CRC.

### GC

GC is a severe cancer worldwide, ranking fifth and fourth globally in incidence and mortality, respectively [63]. In GC, tsRNAs can affect the proliferation and metastasis of GC cells and regulate the development of GC. By small RNA sequencing, Shen et al. found that tRF-33-P4R8YP9LON4VDP was significantly decreased in GC tissues. Quantitative real-time PCR revealed that the expression level of tRF-33-P4R8YP9LON4VDP was decreased in the plasma of GC patients, consistent with the high-throughput sequencing results. Subsequently, cell function experiments showed that this molecule can inhibit the proliferation and migration of GC cells and could promote apoptosis and alter the cell cycle, which demonstrated the potential of tsRNAs to regulate the progression of GC [77]. Another study found that the tRF-3017A derived from tRNA^Val−TAC^ can combine with AGO protein to regulate the expression of cancer suppressor gene NELL2, which promotes migration of GC cells and metastasis of lymph nodes in GC patients, and has the effect of facilitating GC malignant progression [29]. Besides, the expression of tRF-19-3L7L73JD was decreased in GC, which could block the GC cell cycle in the G0/G1 phase, induce apoptosis, inhibit cell proliferation and migration, and thus inhibit the malignant progression of GC [78]. Moreover, tsRNAs can also influence the progression of GC by regulating signaling pathways. tRF-24-V29K9UV3IU was found by Dong et al. to inhibit the Wnt signaling pathway by regulating the proliferation and metastatic ability of GC cells, which in turn hinders the progression of GC [79]. tRF-5026a was found to be able to regulate the PTEN/PI3K/AKT signaling pathway, and upregulation of tRF-5026a could inhibit the proliferation and metastasis of GC cells, which played an influential inhibitory role in the progression of GC also [80]. Xu et al. selected tRF-GluTTC-027 with down-regulated expression for their research, and they found that tRF-GluTTC-027 was associated with the MAPK signaling pathway by GO function and KEGG pathway analysis. Further validation revealed that tRF-GluTTC-027 could significantly regulate the related proteins in the MAPK signaling pathway and play a cancer suppressive role in GC progression [81]. Our research found that tRF-31-U5YKFN8DYDZDD and hsa_tsr016141 could be used as diagnostic and prognostic markers for GC, suggesting that tsRNAs could also be novel biomarkers for GC [82, 83]. As a conclusion, tsRNAs can affect the proliferation and metastasis of GC and regulate the malignant progression of GC through multiple mechanisms. Meanwhile, tsRNAs can also be potential biomarkers for the diagnosis and prognosis of GC, providing new insights and targets for the diagnosis and treatment of GC in clinical practice.

### Hematological tumors

Besides solid tumors, tsRNAs have been reported in hematologic tumors as well. In B-cell lymphomas, low expression of tRF-3 CU1276 could modulate the molecular response to cellular DNA damage and inhibit cell proliferation by targeting replication protein A1(RPA1) [41]. In addition, Guo et al. found that tsRNAs were dysregulated in myelodysplastic syndrome (MDS), and the expression of tsRNAs correlated with whether MDS patients would develop acute myelocytic leukemia (AML) [84]. Chronic lymphocytic leukemia (CLL) is the most common human leukemia, and the expression of ts-3676 is decreased in almost all CLL. It was found that decreased expression levels of ts-3676 diminished the ability to target the 3’-UTR of T cell lymphoma breakpoint 1 (TCL1), leading to increased expression of TCL1, which aggravated the severity of CLL consequently [85]. A follow-up study found that ts-3676 and ts-4521 expression was significantly dysregulated in both LC and CLL, suggesting that tsRNAs play a vital role in both solid and hematologic tumors [86].

### Other cancers

Except for the cancers discussed above, tsRNAs play significant roles in other cancers as well. In hepatocellular carcinoma (HCC), Gly-tRF targets Nedd4 family interacting protein 2 (NDFIP2) to promote the growth of HCC cells. Through analysis, NDFIP2 was found to regulate the AKT signaling pathway via ubiquitination of downstream target proteins, thereby regulating the malignant progression of HCC [87]. The expression of tRF-315 was increased in prostate cancer (PC) cells and could inhibit cisplatin-induced apoptosis. In LNCaP and DU145 cells, tRF-315 could also alleviate cisplatin-induced mitochondrial dysfunction. Further study revealed that tRF-315 protected PC cells from cisplatin-induced mitochondrial-dependent apoptosis by targeting the tumor suppressor gene (GADD45A) [88]. In LC, the expression of ts-46 and ts-47 was decreased and inhibited the growth of LC cells, suggesting that tsRNAs may be involved in cancer pathogenesis and are key substances in cancer-related pathways [89]. tRF-Leu-CAG can promote proliferation and cell cycle in non-small cell LC (NSCLC) cells. Follow-up studies revealed that tRF-Leu-CAG might target AURKA to participate in some signaling pathways to perform its cancer-promoting effects. Still, the exact mechanism remains further validated later [90]. Furthermore, the upregulation of tsRNA-5001a is associated with poor prognosis, promoting lung adenocarcinoma (LUAD) cell proliferation and increasing the risk of postoperative recurrence in LUAD patients [91]. In papillary thyroid carcinoma (PTC), highly expressed tiRNA-Gly can directly bind to RNA binding motif protein 17 (RBM17) and lead to RBM17-dependent phosphorylation of downstream signaling pathways by inducing mitogen-activated protein kinase 4 (MAP4K4) mRNA exon 16 splicing. Subsequent transfer of RBM17 from the cytoplasm to the nucleus increases RBM17 protein expression by inhibiting RBM17 degradation, promoting PTC progression [92].

Taken together, tsRNAs play an essential role in various types of cancer, they can regulate multiple signaling pathways and have a complex regulatory relationship with tumor biogenesis. With the continuous improvement of the tsRNAs database and detection technology, it is believed that tsRNAs will be applied to clinical applications and provide a new way of thinking for tumor treatment.

## Clinical application value of tsRNAs in cancer: as biomarkers for cancer diagnosis and prognosis

As a non-invasive and convenient sampling technique for tumor diagnosis, liquid biopsy has gradually replaced invasive methods for cancer diagnosis and detection in the last decade [93]. Meanwhile, an increasing number of studies have found that tsRNAs are present not only in the tissues, but also widely in the body fluids of cancer patients, including plasma, serum, and exosomes. It has been reported that the expression of tsRNAs differs significantly between cancer patients and normal controls, correlates with clinicopathological parameters of patients, and can predict the poor prognosis of patients. If tsRNAs are well integrated with liquid biopsy techniques and applied in the clinical practice, it is believed that they will provide new biomarkers for the clinical diagnosis of cancers and open up new avenues for the detection of cancer patients [94] (Table 1).

**Table 1:** Clinical application value of tsRNAs in cancer

Source	tsRNAs name	tsRNAs type	Cancer type	Expression	Clinical significance	References
Plasm	tiRNA-5034-GluTTC-2	5′tiRNA	GC	Down	Have good diagnostic efficacy for GC, which was further improved by combining their expression levels in tissues and plasma	[95]
	tRF-33-P4R8YP9LON4VDP	5′tiRNA	GC	Down	Serve as a novel diagnostic biomarker and target for GC therapeutics	[77]
	tRF-19-3L7L73JD	i-tRF	GC	Down	Associated with tumor size and may be useful as a biomarker of GC	[78]
	tRF-5026a	tRF-5	GC	Down	Related to tumor size and has certain diagnostic value to predict the overall survival	[80]
	tRF-Glu-CTC-003, tRF-Gly-CCC-007, tRF-Gly-CCC-008	tRF-5	EBC	Down	Serve as novel biomarkers for the diagnosis of EBC	[107]
	tRF-Leu-CAA-003, tRF-Ser-TGA-001, tRF-Ser-TGA-002					
	tRF-Gly-CCC-001	tRF-1	BC	Up	Serve as diagnostic biomarkers for BC	[96]
	tRF-Arg-CCT-017, tiRNA-Phe-GAA-003	tRF-1, 5′tiRNA	BC	Up	Serve as diagnostic and prognostic markers of BC	
	tRF-16-PSQP4PE, tRF-21-RK9P4P9L0	tRF-5	LC	Up	Combining the three tsRNAs may predict LUAD	[97]
	tRF-16-L85J3KE	i-tRF	LC	Down		
	5’-tRF-GlyGCC	tRF-5	CRC	Up	Serve as a promising diagnostic biomarker for CRC diagnosis	[98]
Serum	hsa_tsr016141	tRF-5	GC	Up	Serve as a diagnostic marker for GC and dynamically monitor the postoperative situation	[82]
	tRF-31-U5YKFN8DYDZDD	i-tRF	GC	Up	Related to TNM stage, depth of tumor invasion, lymph node metastasis, and vascular invasion	[83]
	tRF-Leu-CAG	5′tiRNA	NSCLC	Up	Significantly correlated with tumor progression	[90]
	tDR‐000,620	5′tiRNA	TNBC	Down	May provide predictive biomarkers and therapeutic targets for TNBC recurrence	[99]
	5′-tiRNA^Val^	5'tiRNA	BC	Down	Positively correlated with stage progression and lymph node metastasis	[65]
	tRF-30-JZOYJE22RR33, tRF-27-ZDXPHO53KSN	tRF-3	BC	Up	Correlated with trastuzumab resistance	[100]
	tRF-17-79MP9PP	tRF-5	BC	Down	Serve as novel biomarkers for the diagnosis of EBC	[102]
	tDR-7816	i-tRF	BC	Down	Potential diagnostic biomarker for patients with early non-TNBC	[101]
	tsRNA-MetCAT-37, tsRNA-ValTAC-41, tsRNA-ThrTGT-23	tRF-3	PDAC	Up	may be promising and effective candidates for PDAC diagnosis	[108]
	tRF-Gln-TTG-006	tRF-5	HCC	Up	can distinguish between HCC patients and healthy controls even in early stages	[103]
Exosome	tRF-38, tRF-18	tRF-3	GC	Up	discriminate GC patients with a high risk of recurrence and poor prognosis	[104]
	tRF-25	i-tRF				
	tRNA-ValTAC-3	tRF-3	HCC	Up	serve as a novel “liquid biopsy” biomarker for HCC diagnosis	[105]
	tRNA-GlyTCC-5, tRNA-ValAAC-5	5'tiRNA				
	tRNA-GluCTC-5	tRF-5				
	tRNA-GlyGCC-5	tRF-5	ESCC	Up	predict of ESCC patients who likely to benefit from adjuvant therapy	[106]

In terms of plasma, Zhu et al. first found that the expression level of tiRNA-5034-GluTTC-2 was significantly downregulated in both tissues and plasma of GC patients. By receiver operating characteristic analysis, they found that tiRNA-5034-GluTTC-2 had better diagnostic efficacy, which was further increased by combining the expression levels in tissues and plasma of it, indicating that tiRNA-5034-GluTTC-2 has the potential to become a biomarker for GC [95]. Wang et al. found that the expression levels of tRF-Arg-CCT-017, tRF-Gly-CCC-001, and tiRNA-Phe-GAA-003 were significantly increased in the plasma of BC patients by high-throughput sequencing, while the expression levels of tRF-Gly-CCC-001 and tiRNA-Phe-GAA-003 in exosomes correlated significantly with the expression levels in plasma, indicating that most of them in plasma were also present in exosomes [96]. In LC, Wang et al. found that the expression of tRF-16-L85J3KE was downregulated in the plasma of LUAD patients, while the expression of tRF-21-RK9P4P9L0 and tRF-16-PSQP4PE was upregulated, consistent with the RNA sequencing results. The Area Under Curve (AUC) of these three tsRNAs reached 0.92 when they were combined to diagnose LUAD, suggesting good diagnostic efficacy. Additionally, tRF-21-RK9P4P9L0 was negatively correlated with LUAD prognosis, and inhibition of tRF-21-RK9P4P9L0 expression diminished the proliferation, migration, and invasive ability of LC cell lines [97]. In the plasma of CRC patients, the expression level of 5-tRF-GlyGCC was significantly elevated with an AUC of 0.882 and was further increased to 0.926 after combined with carcinoembryonic antigen and Carbohydrate antigen199, suggesting its good diagnostic efficacy for CRC [98].

On the serum side, the expression levels of hsa_tsr016141 and tRF-31-U5YKFN8DYDZDD were significantly increased in the serum of GC patients, which could effectively distinguish GC patients from normal controls and their expression levels were significantly decreased after GC surgery. Moreover, they were both good diagnostic markers and predictors of poor prognosis in GC [82, 83]. Upregulated tRF-Leu-CAG in NSCLC serum was significantly associated with tumor progression, while tRF-Leu-CAG expression was similarly upregulated in NSCLC cell lines. Inhibition of tRF-Leu-CAG suppressed cell proliferation and impeded the cell cycle, suggesting that it may be a new diagnostic marker and potential therapeutic target in NSCLC [90]. Significantly decreased tDR-000620 in the serum of TNBC patients, which was associated with lymph node status and recurrence, was an independent poor predictor of recurrence-free survival, and its expression level may be a factor in the recurrence and poor prognosis of TNBC patients [99]. In addition, the expression levels of tRF-30-JZOYJE22RR33 and tRF-27-ZDXPHO53KSN were significantly elevated in the serum of trastuzumab-resistant BC patients, and patients with high expression levels of both benefited to a lesser extent from trastuzumab-based therapy, suggesting that both of them play an important role in trastuzumab resistance and could be clinical targets for the treatment of trastuzumab-resistant BC as an intervention target and potential biomarker [100]. Besides, tDR-7816 was significantly differentially expressed in the serum of non-TNBC patients and may affect xenobiotic metabolic processes associated with breast carcinogenesis [101]. The expression levels of 5′-tiRNA^Val^ and tRF-17-79MP9PP were significantly decreased in the serum of BC patients, and both were significantly correlated, with 5′-tiRNA^Val^ being associated with TNM stage and lymph node metastasis [65, 102]. In addition, tRF-Gln-TTG-006 derived from tumor cells was found to be significantly upregulated in HCC serum, and the signature of tRF-Gln-TTG-006 could distinguish HCC patients from healthy controls, even when HCC patients were in early stages (sensitivity: 79.0%; specificity: 74.8%) [103].

From the exosome aspect, tRF-38, tRF-25, and tRF-18 were expressed elevated in plasma exosomes of GC patients, which has good accuracy for GC diagnosis and may be a predictor of GC after surgery [104]. Besides, the expression levels of tRNA-ValTAC-3, tRNA-GlyTCC-5, tRNA-ValAAC-,5, and tRNA-GluCTC-5 were significantly increased in plasma exosomes from patients with HCC, indicating that plasma exosomal tsRNAs could be new diagnostic biomarkers [105]. The tRNA-GlyGCC-5, derived from salivary exosomes, was a noninvasive biomarker for the diagnosis and prognosis of human Esophageal squamous cell carcinoma (ESCC) and could predict preoperative patients who might benefit from adjuvant therapy [106].

In summary, tsRNAs have shown promising biomarker potential and clinical application in plasma, serum, and exosomes of cancer patients. The detection of tsRNAs expression in body fluids of cancer patients is expected to be a new and better technique applicable to the noninvasive diagnosis of cancer in the future.

## Databases for tsRNAs

There are many databases for tsRNAs that can boost the research of tsRNAs (Table 2). tRFdb is the earliest database about tRFs, which contains information about tRFs in eight species and can be set for specific species as well as searched by tRF ID or tRF sequence [50]. PtRFdb includes various information about tRFs in plants, facilitating the study of tRFs in plants [109]. tRex is a database of tRFs in plants Arabidopsis thaliana and summarizes information about the sequencing of multiple small RNAs, which conveniently facilitates the study of tRFs in Arabidopsis thaliana [110]. MINTbase v2.0 was updated based on the first generation MINTbase, containing information about tRFs in a variety of human tissues. It can be searched to show the abundance of tRFs in various cancers on demand [111]. Apart from that, tRFs gene network prediction, tRFs survival analysis, and functional enrichment analysis can be performed in OncotRF according to the settings of users [112].

**Table 2:** Databases for tsRNAs

Name	Function	Website
MINTbase	Contains basic information about tRFs currently found in a variety of human tissues and demonstrates the abundance of tRFs in a variety of cancers	http://cm.jefferson.edu/MINTbase/
OncotRF	Can be used to search the expression level and related information of corresponding tRFs in different cancers, and has the functions of tRFs gene network, functional enrichment analysis and survival analysis	http://bioinformatics.zju.edu.cn/OncotRF
PtRFdb	Contains detailed information about tRFs in different plants, is a database of tRFs in plants, helpful for the study of tRFs in plants	http://www.nipgr.res.in/PtRFdb/
tRex	Database of tRFs studies in arabidopsis thaliana	http://combio.pl/trex
tRFdb	Contains information on 3 tRFs for 8 species, searchable by tRF sequence or tRF ID	http://genome.bioch.virginia.edu/trfdb/
tRFexplorer	Differential expression of tsRNAs in TCGA in NCI-60 can be analyzed, and correlation analysis can be performed with genes or miRNAs in TCGA samples	https://trfexplorer.cloud/
tRFTar	Can be customized to search the interaction relationship between tRFs and target genes, and has enrichment analysis and other functions, which is helpful to explore the function of tRFs	http://www.rnanut.net/tRFTar/
tRFTars	The first database to predict potential tRFs targets in humans	http://trftars.cmuzh.ennin.glab.org:3838/tar/
tsRBase	Contains the basic characteristics of tsRNAs in different species as well as the target and biological function information of tsRNAs, which can be searched according to research needs	http://www.tsrbase.org
tsRFun	Provides tsRNAs identification, target prediction, enrichment analysis and other online tools	http://rna.sysu.edu.cn/tsRFun/orhttp://biomed.nscc-gz.cn/DB/tsRFun/
tRFtarget	RNAhybrid and IntaRNA can be used to predict tRFs targets by predicting the interaction between tRFs and transcripts	http://trftarget.net

Besides providing basic information on tsRNAs, the following databases help study tsRNAs-related functions and mechanisms. tRFTar can be customized to search and filter the interaction of tRFs with target genes and can perform enrichment analysis of the functions of tRFs [113]. tRFTars can predict the potential target genes of tRFs online and contribute to studying the function and mechanism of tRFs [114]. tsRFun database contains the expression profiles of tsRNAs in 32 cancers, which enables online prediction of the targets of tsRNAs and the construction of interaction networks with mRNAs [115]. tRFtarget database provides a comprehensive prediction of the interaction between tRFs and targets through two advanced tools, RNAhybrid and IntaRNA, and allows the analysis of the predicted target genes [116]. tsRBase contains tsRNAs from different species and different types of samples information, which also provides valuable information on tsRNAs and basic information. Also, tsRBase collected the latest literature on tsRNAs research, which provides a comprehensive aid for studying tsRNAs [117].

## Conclusions

With the in-depth investigation of tsRNAs, the biological functions of tsRNAs such as regulation of gene expression, translation, and epigenetics have been gradually revealed. tsRNAs dysregulation is closely related to the development of various cancers. At the same time, dysregulated tsRNAs have the potential to act as tumor biomarkers and can provide new targets for cancer diagnosis and treatment. Moreover, they can dynamically monitor cancer patients and provide new targets for cancer diagnosis and treatment. Currently, databases of tsRNAs are emerging, covering tsRNAs from different species and different types of samples, which can provide researchers with basic information on the origin and sequence of tsRNAs. In addition, many databases also have the function of predicting the targets of tsRNAs, which contributes to the study of the molecular mechanism of tsRNAs and related processes. The emergence of these databases has promoted the rapid development of tsRNAs research.

However, although tsRNAs are currently found to be dysregulated in a variety of cancers, the mechanisms by which they genuinely influence cancer progression remain unclear, and studies on the functions and mechanisms of tsRNAs are still inadequate. More in-depth molecular mechanisms of tsRNAs still need further exploration and discovery in the future. In addition, studies on tsRNAs as tumor diagnostic and prognostic markers are still in their infancy, and the sample size of studies on whether they can be applied to clinical analysis still needs to be expanded. Meanwhile, the number of tsRNAs included in many databases related to the functions of tsRNAs is still limited, many tsRNAs cannot be analyzed, so it is still necessary to expand the number of tsRNAs included in databases in the future.

In conclusion, studies on tsRNAs have been emerging in recent years, with an increasing number of tsRNAs and their research tools have been discovered and applied. It is believed that in the future, with the advancement of technology, the role and mechanism of tsRNAs in cancer will also become increasingly apparent. As an emerging non-coding small RNA, the emergence of tsRNAs provides a new direction and possibility for diagnosing and treating cancer in the future.

## Data Availability

Not applicable.

## Abbreviations

tRNAs: Transfer RNAs
sncRNA: Small non-coding RNA
Pol III: RNA polymerase III
pre-tRNAs: Precursor tRNAs
tsRNAs: TRNA-derived small RNAs
tRFs: TRNA-derived fragments
tiRNAs: TRNA halves
ANG: Angiopoietin
SHOT-RNAs: Sex hormone-dependent tRNA-derived RNAs
miRNAs: MicroRNAs
BC: Breast cancer
sitRNA-3: Stress-induced tRNA-3
sitRNA-5: Stress-induced tRNA-5
RISC: RNA-induced silencing complex
3′-UTR: 3′-Untranslated region
GC: Gastric cancer
AGO: Argonaute
NELL2: Neural EGFL like 2
RBP: RNA-binding protein
YBX1: Y-box binding protein 1
IGF2BP1: Insulin like growth factor 2 mRNA binding protein 1
mESCs: Mouse embryonic stem cells
HIV-1: Human immunodeficiency virus-1
PBS: Primer-binding site
RSV: Respiratory syncytial virus
RG4: RNA G-quadruplexes
RPS28: Ribosomal protein S28
CDS: Coding sequence
piRNAs: PIWI-interacting RNAs
DNMT2: DNA methyltransferase-2
CRC: Colorectal cancer
LC: Lung cancer
NCL: Nucleolin
FZD3: Frizzled class receptor 3
RPL27A: Ribosomal protein L27a
RUNX1: RUNX family transcription factor 1
TNBC: Triple negative BC
G6PC: Glucose-6-phosphatase catalytic
RPA1: Replication protein A1
MDS: Myelodysplastic syndrome
AML: Acute myelocytic leukemia
CLL: Chronic lymphocytic leukemia
TCL1: T cell lymphoma breakpoint 1
HCC: Hepatocellular carcinoma
NDFIP2: Nedd4 family interacting protein 2
PC: Prostate cancer
GADD45A: Growth arrest and DNA damage inducible alpha
NSCLC: Non-small cell LC
LUAD: Lung adenocarcinoma
PTC: Papillary thyroid carcinoma
RBM17: RNA binding motif protein 17
MAP4K4: Mitogen-activated protein kinase 4
AUC: Area Under Curve
CA199: Carbohydrate antigen199
ESCC: Esophageal squamous cell carcinoma

## References

[1] Huang S, Sun B, Xiong Z, Shu Y, Zhou H, Zhang W, Xiong J, Li Q (2018). The dysregulation of tRNAs and tRNA derivatives in cancer. J Exp Clin Cancer Res.

[2] Schaffer AE, Pinkard O, Coller JM (2019). tRNA metabolism and neurodevelopmental disorders. Annu Rev Genom Hum Genet.

[3] Zhu C, Sun B, Nie A, Zhou Z (2020). The tRNA-associated dysregulation in immune responses and immune diseases. Acta Physiol.

[4] Shen Y, Yu X, Zhu L, Li T, Yan Z, Guo J (2018). Transfer RNA-derived fragments and tRNA halves: biogenesis, biological functions and their roles in diseases. J Mol Med (Berl).

[5] Giegé R (2008). Toward a more complete view of tRNA biology. Nat Struct Mol Biol.

[6] Rodnina MV, Wintermeyer W (2011). The ribosome as a molecular machine: the mechanism of tRNA-mRNA movement in translocation. Biochem Soc Trans.

[7] Kim HK, Yeom JH, Kay MA (2020). Transfer RNA-derived small RNAs: another layer of gene regulation and novel targets for disease therapeutics. Mol Ther.

[8] Borek E, Baliga B, Gehrke C, Kuo C, Belman S, Troll W, Waalkes T (1977). High turnover rate of transfer RNA in tumor tissue. Cancer Res.

[9] Speer J, Gehrke CW, Kuo KC, Waalkes TP, Borek E (1979). tRNA breakdown products as markers for cancer. Cancer.

[10] Zhu L, Ge J, Li T, Shen Y, Guo J (2019). tRNA-derived fragments and tRNA halves: the new players in cancers. Cancer Lett.

[11] Xie Y, Yao L, Yu X, Ruan Y, Li Z, Guo J (2020). Action mechanisms and research methods of tRNA-derived small RNAs. Signal Transduct Target Ther.

[12] Couvillion M, Sachidanandam R, Collins K (2010). A growth-essential Tetrahymena Piwi protein carries tRNA fragment cargo. Genes Dev.

[13] Zhu P, Yu J, Zhou P (2020). Role of tRNA-derived fragments in cancer: novel diagnostic and therapeutic targets tRFs in cancer. Am J Cancer Res.

[14] Honda S, Loher P, Shigematsu M, Palazzo JP, Suzuki R, Imoto I, Rigoutsos I, Kirino Y (2015). Sex hormone-dependent tRNA halves enhance cell proliferation in breast and prostate cancers. Proc Natl Acad Sci USA.

[15] Katsaraki K, Artemaki PI, Papageorgiou SG, Pappa V, Scorilas A, Kontos CK (2019). Identification of a novel, internal tRNA-derived RNA fragment as a new prognostic and screening biomarker in chronic lymphocytic leukemia, using an innovative quantitative real-time PCR assay. Leuk Res.

[16] Kumar P, Anaya J, Mudunuri S, Dutta A (2014). Meta-analysis of tRNA derived RNA fragments reveals that they are evolutionarily conserved and associate with AGO proteins to recognize specific RNA targets. BMC Biol.

[17] Zhu LW, Xie Y, Guo JM (2017). The biological functions of tRNA-derived fragments and tRNA halves, and their roles in the pathogenesis. Prog Biochem Biophysics.

[18] Cole C, Sobala A, Lu C, Thatcher SR, Bowman A, Brown JW, Green PJ, Barton GJ, Hutvagner G (2009). Filtering of deep sequencing data reveals the existence of abundant Dicer-dependent small RNAs derived from tRNAs. RNA.

[19] Park EJ, Kim TH (2018). Fine-tuning of gene expression by tRNA-derived fragments during abiotic stress signal transduction. Int J Mol Sci.

[20] Goodarzi H, Liu X, Nguyen HC, Zhang S, Fish L, Tavazoie SF (2015). Endogenous tRNA-derived fragments suppress breast cancer progression via YBX1 displacement. Cell.

[21] Kumar P, Kuscu C, Dutta A (2016). Biogenesis and function of transfer RNA-related fragments (tRFs). Trends Biochem Sci.

[22] Li S, Xu Z, Sheng J (2018). tRNA-derived small RNA: a novel regulatory small non-coding RNA. Genes.

[23] Li S, Hu GF (2012). Emerging role of angiogenin in stress response and cell survival under adverse conditions. J Cell Physiol.

[24] Li S, Shi X, Chen M, Xu N, Sun D, Bai R, Chen H, Ding K, Sheng J, Xu Z (2019). Angiogenin promotes colorectal cancer metastasis via tiRNA production. Int J Cancer.

[25] Anderson P, Ivanov P (2014). tRNA fragments in human health and disease. FEBS Lett.

[26] Saikia M, Hatzoglou M (2015). The many virtues of tRNA-derived stress-induced RNAs (tiRNAs): discovering novel mechanisms of stress response and effect on human health. J Biol Chem.

[27] Li Y, Luo J, Zhou H, Liao JY, Ma LM, Chen YQ, Qu LH (2008). Stress-induced tRNA-derived RNAs: a novel class of small RNAs in the primitive eukaryote *Giardia*
*lamblia*. Nucleic Acids Res.

[28] He L, Hannon GJ (2004). MicroRNAs: small RNAs with a big role in gene regulation. Nat Rev Genet.

[29] Tong L, Zhang W, Qu B, Zhang F, Wu Z, Shi J, Chen X, Song Y, Wang Z (2020). The tRNA-derived fragment-3017A promotes metastasis by inhibiting NELL2 in human gastric cancer. Front Oncol.

[30] Choi EJ, Ren J, Zhang K, Wu W, Lee YS, Lee I, Bao X (2020). The Importance of AGO 1 and 4 in post-transcriptional gene regulatory function of trf5-gluctc, an respiratory syncytial virus-induced tRNA-derived RNA fragment. Int J Mol Sci.

[31] Cao KY, Yan TM, Zhang JZ, Chan TF, Li J, Li C, Lai-Han Leung E, Gao J, Zhang BX, Jiang ZH (2022). A tRNA-derived fragment from Chinese yew suppresses ovarian cancer growth via targeting TRPA1. Mol Ther Nucleic Acids.

[32] Li J, Zhu L, Cheng J, Peng Y (2021). Transfer RNA-derived small RNA: a rising star in oncology. Semin Cancer Biol.

[33] Krishna S, Yim DG, Lakshmanan V, Tirumalai V, Koh JL, Park JE, Cheong JK, Low JL, Lim MJ, Sze SK (2019). Dynamic expression of tRNA-derived small RNAs define cellular states. EMBO Rep.

[34] Yeung ML, Bennasser Y, Watashi K, Le SY, Houzet L, Jeang KT (2009). Pyrosequencing of small non-coding RNAs in HIV-1 infected cells: evidence for the processing of a viral-cellular double-stranded RNA hybrid. Nucleic Acids Res.

[35] Wang Q, Lee I, Ren J, Ajay SS, Lee YS, Bao X (2013). Identification and functional characterization of tRNA-derived RNA fragments (tRFs) in respiratory syncytial virus infection. Mol Ther.

[36] Ruggero K, Guffanti A, Corradin A, Sharma VK, De Bellis G, Corti G, Grassi A, Zanovello P, Bronte V, Ciminale V, D'Agostino DM (2014). Small noncoding RNAs in cells transformed by human T-cell leukemia virus type 1: a role for a tRNA fragment as a primer for reverse transcriptase. J Virol.

[37] Deng J, Ptashkin RN, Chen Y, Cheng Z, Liu G, Phan T, Deng X, Zhou J, Lee I, Lee YS, Bao X (2015). Respiratory syncytial virus utilizes a tRNA fragment to suppress antiviral responses through a novel targeting mechanism. Mol Ther.

[38] Shi J, Zhang Y, Zhou T, Chen Q (2019). tsRNAs: the Swiss army knife for translational regulation. Trends Biochem Sci.

[39] Gebetsberger J, Wyss L, Mleczko AM, Reuther J, Polacek N (2017). A tRNA-derived fragment competes with mRNA for ribosome binding and regulates translation during stress. RNA Biol.

[40] Luo S, He F, Luo J, Dou S, Wang Y, Guo A, Lu J (2018). Drosophila tsRNAs preferentially suppress general translation machinery via antisense pairing and participate in cellular starvation response. Nucleic Acids Res.

[41] Maute RL, Schneider C, Sumazin P, Holmes A, Califano A, Basso K, Dalla-Favera R (2013). tRNA-derived microRNA modulates proliferation and the DNA damage response and is down-regulated in B cell lymphoma. Proc Natl Acad Sci USA.

[42] Ren B, Wang X, Duan J, Ma J (2019). Rhizobial tRNA-derived small RNAs are signal molecules regulating plant nodulation. Science.

[43] Kim HK, Fuchs G, Wang S, Wei W, Zhang Y, Park H, Roy-Chaudhuri B, Li P, Xu J, Chu K (2017). A transfer-RNA-derived small RNA regulates ribosome biogenesis. Nature.

[44] Kim HK, Xu J, Chu K, Park H, Jang H, Li P, Valdmanis PN, Zhang QC, Kay MA (2019). A tRNA-derived small RNA regulates ribosomal protein S28 protein levels after translation initiation in humans and mice. Cell Rep.

[45] Lyons SM, Gudanis D, Coyne SM, Gdaniec Z, Ivanov P (2017). Identification of functional tetramolecular RNA G-quadruplexes derived from transfer RNAs. Nat Commun.

[46] Akiyama Y, Kharel P, Abe T, Anderson P, Ivanov P (2020). Isolation and initial structure-functional characterization of endogenous tRNA-derived stress-induced RNAs. RNA Biol.

[47] Yan Q, Zhu C, Guang S, Feng X (2019). The functions of non-coding RNAs in rRNA regulation. Front Genet.

[48] Couvillion MT, Bounova G, Purdom E, Speed TP, Collins K (2012). A Tetrahymena Piwi bound to mature tRNA 3′ fragments activates the exonuclease Xrn2 for RNA processing in the nucleus. Mol Cell.

[49] Fricker R, Brogli R, Luidalepp H, Wyss L, Fasnacht M, Joss O, Zywicki M, Helm M, Schneider A, Cristodero M, Polacek N (2019). A tRNA half modulates translation as stress response in Trypanosoma brucei. Nat Commun.

[50] Kumar P, Mudunuri SB, Anaya J, Dutta A (2015). tRFdb: a database for transfer RNA fragments. Nucleic Acids Res.

[51] Thomson T, Lin H (2009). The biogenesis and function of PIWI proteins and piRNAs: progress and prospect. Annu Rev Cell Dev Biol.

[52] Lin H, Spradling AC (1997). A novel group of pumilio mutations affects the asymmetric division of germline stem cells in the Drosophila ovary. Development.

[53] Pei H, Yao Y, Yang Y, Liao K, Wu JR (2011). Krüppel-like factor KLF9 regulates PPARγ transactivation at the middle stage of adipogenesis. Cell Death Differ.

[54] Lee H, Kim HJ, Lee YJ, Lee MY, Choi H, Lee H, Kim JW (2012). Krüppel-like factor KLF8 plays a critical role in adipocyte differentiation. PLoS ONE.

[55] Zhang Y, Shi J, Chen Q (2017). tsRNAs: new players in mammalian retrotransposon control. Cell Res.

[56] Perez MF, Lehner B (2019). Intergenerational and transgenerational epigenetic inheritance in animals. Nat Cell Biol.

[57] Chen Q, Yan M, Cao Z, Li X, Zhang Y, Shi J, Feng GH, Peng H, Zhang X, Zhang Y (2016). Sperm tsRNAs contribute to intergenerational inheritance of an acquired metabolic disorder. Science.

[58] Jacovetti C, Bayazit MB, Regazzi R (2021). Emerging classes of small non-coding RNAs with potential implications in diabetes and associated metabolic disorders. Front Endocrinol.

[59] Siomi MC, Sato K, Pezic D, Aravin AA (2011). PIWI-interacting small RNAs: the vanguard of genome defence. Nat Rev Mol Cell Biol.

[60] Martienssen R, Moazed D (2015). RNAi and heterochromatin assembly. Cold Spring Harb Perspect Biol.

[61] Sharma U, Conine CC, Shea JM, Boskovic A, Derr AG, Bing XY, Belleannee C, Kucukural A, Serra RW, Sun F (2016). Biogenesis and function of tRNA fragments during sperm maturation and fertilization in mammals. Science.

[62] Komarov PA, Sokolova O, Akulenko N, Brasset E, Jensen S, Kalmykova A (2020). Epigenetic Requirements for triggering heterochromatinization and piwi-interacting RNA production from transgenes in the drosophila germline. Cells.

[63] Sung H, Ferlay J, Siegel R, Laversanne M, Soerjomataram I, Jemal A, Bray F (2021). Global cancer statistics 2020: GLOBOCAN estimates of incidence and mortality worldwide for 36 cancers in 185 countries. CA Cancer J Clin.

[64] Falconi M, Giangrossi M, Zabaleta ME, Wang J, Gambini V, Tilio M, Bencardino D, Occhipinti S, Belletti B, Laudadio E (2019). A novel 3′-tRNA(Glu)-derived fragment acts as a tumor suppressor in breast cancer by targeting nucleolin. FASEB J.

[65] Mo D, Jiang P, Yang Y, Mao X, Tan X, Tang X, Wei D, Li B, Wang X, Tang L, Yan F (2019). A tRNA fragment, 5′-tiRNA(Val), suppresses the Wnt/β-catenin signaling pathway by targeting FZD3 in breast cancer. Cancer Lett.

[66] Zhang Z, Liu Z, Zhao W, Zhao X, Tao Y (2022). tRF-19-W4PU732S promotes breast cancer cell malignant activity by targeting inhibition of RPL27A (ribosomal protein-L27A). Bioengineered.

[67] Farina NH, Scalia S, Adams CE, Hong D, Fritz AJ, Messier TL, Balatti V, Veneziano D, Lian JB, Croce CM (2020). Identification of tRNA-derived small RNA (tsRNA) responsive to the tumor suppressor, RUNX1, in breast cancer. J Cell Physiol.

[68] Shan N, Li N, Dai Q, Hou L, Yan X, Amei A, Lu L, Wang Z (2020). Interplay of tRNA-derived fragments and T cell activation in breast cancer patient survival. Cancers.

[69] Telonis AG, Rigoutsos I (2018). Race disparities in the contribution of miRNA isoforms and tRNA-derived fragments to triple-negative breast cancer. Cancer Res.

[70] Cui Y, Huang Y, Wu X, Zheng M, Xia Y, Fu Z, Ge H, Wang S, Xie H (2019). Hypoxia-induced tRNA-derived fragments, novel regulatory factor for doxorubicin resistance in triple-negative breast cancer. J Cell Physiol.

[71] Zhu P, Lu J, Zhi X, Zhou Y, Wang X, Wang C, Gao Y, Zhang X, Yu J, Sun Y, Zhou P (2021). tRNA-derived fragment tRFLys-CTT-010 promotes triple-negative breast cancer progression by regulating glucose metabolism via G6PC. Carcinogenesis.

[72] Tao EW, Wang HL, Cheng WY, Liu QQ, Chen YX, Gao QY (2021). A specific tRNA half, 5'tiRNA-His-GTG, responds to hypoxia via the HIF1α/ANG axis and promotes colorectal cancer progression by regulating LATS2. J Exp Clin Cancer Res.

[73] Luan N, Mu Y, Mu J, Chen Y, Ye X, Zhou Q, Xu M, Deng Q, Hu Y, Tang Z, Wang J (2021). Dicer1 promotes colon cancer cell invasion and migration through modulation of tRF-20-MEJB5Y13 expression under hypoxia. Front Genet.

[74] Huang B, Yang H, Cheng X, Wang D, Fu S, Shen W, Zhang Q, Zhang L, Xue Z, Li Y (2017). tRF/miR-1280 suppresses stem cell-like cells and metastasis in colorectal cancer. Cancer Res.

[75] Chen H, Xu Z, Cai H, Peng Y, Yang L, Wang Z (2022). Identifying differentially expressed tRNA-derived small fragments as a biomarker for the progression and metastasis of colorectal cancer. Dis Markers.

[76] Wang X, Zhang Y, Ghareeb WM, Lin S, Lu X, Huang Y, Huang S, Xu Z, Chi P (2020). A comprehensive repertoire of transfer RNA-derived fragments and their regulatory networks in colorectal cancer. J Comput Biol.

[77] Shen Y, Yu X, Ruan Y, Li Z, Xie Y, Yan Z, Guo J (2021). Global profile of tRNA-derived small RNAs in gastric cancer patient plasma and identification of tRF-33-P4R8YP9LON4VDP as a new tumor suppressor. Int J Med Sci.

[78] Shen Y, Xie Y, Yu X, Zhang S, Wen Q, Ye G, Guo J (2021). Clinical diagnostic values of transfer RNA-derived fragment tRF-19-3L7L73JD and its effects on the growth of gastric cancer cells. J Cancer.

[79] Dong X, Fan X, He X, Chen S, Huang W, Gao J, Huang Y, Wang H (2020). Comprehensively identifying the key tRNA-derived fragments and investigating their function in gastric cancer processes. Onco Targets Ther.

[80] Zhu L, Li Z, Yu X, Ruan Y, Shen Y, Shao Y, Zhang X, Ye G, Guo J (2021). The tRNA-derived fragment 5026a inhibits the proliferation of gastric cancer cells by regulating the PTEN/PI3K/AKT signaling pathway. Stem Cell Res Ther.

[81] Xu W, Zhou B, Wang J, Tang L, Hu Q, Wang J, Chen H, Zheng J, Yan F, Chen H (2021). tRNA-derived fragment tRF-Glu-TTC-027 regulates the progression of gastric carcinoma via MAPK signaling pathway. Front Oncol.

[82] Gu X, Ma S, Liang B, Ju S (2021). Serum hsa_tsr016141 as a kind of tRNA-derived fragments is a novel biomarker in gastric cancer. Front Oncol.

[83] Huang Y, Zhang H, Gu X, Qin S, Zheng M, Shi X, Peng C, Ju S (2021). Elucidating the role of serum tRF-31-U5YKFN8DYDZDD as a novel diagnostic biomarker in gastric cancer (GC). Front Oncol.

[84] Guo Y, Strickland SA, Mohan S, Li S, Bosompem A, Vickers KC, Zhao S, Sheng Q, Kim AS (2017). MicroRNAs and tRNA-derived fragments predict the transformation of myelodysplastic syndromes to acute myeloid leukemia. Leuk Lymphoma.

[85] Balatti V, Rizzotto L, Miller C, Palamarchuk A, Fadda P, Pandolfo R, Rassenti LZ, Hertlein E, Ruppert AS, Lozanski A (2015). TCL1 targeting miR-3676 is codeleted with tumor protein p53 in chronic lymphocytic leukemia. Proc Natl Acad Sci USA.

[86] Pekarsky Y, Balatti V, Palamarchuk A, Rizzotto L, Veneziano D, Nigita G, Rassenti LZ, Pass HI, Kipps TJ, Liu CG, Croce CM (2016). Dysregulation of a family of short noncoding RNAs, tsRNAs, in human cancer. Proc Natl Acad Sci USA.

[87] Zhou Y, Hu J, Liu L, Yan M, Zhang Q, Song X, Lin Y, Zhu D, Wei Y, Fu Z (2021). Gly-tRF enhances LCSC-like properties and promotes HCC cells migration by targeting NDFIP2. Cancer Cell Int.

[88] Yang C, Lee M, Song G, Lim W (2021). tRNA(Lys)-derived fragment alleviates cisplatin-induced apoptosis in prostate cancer cells. Pharmaceutics.

[89] Balatti V, Nigita G, Veneziano D, Drusco A, Stein GS, Messier TL, Farina NH, Lian JB, Tomasello L, Liu CG (2017). tsRNA signatures in cancer. Proc Natl Acad Sci USA.

[90] Shao Y, Sun Q, Liu X, Wang P, Wu R, Ma Z (2017). tRF-Leu-CAG promotes cell proliferation and cell cycle in non-small cell lung cancer. Chem Biol Drug Des.

[91] Hu F, Niu Y, Mao X, Cui J, Wu X, Simone CB, Kang HS, Qin W, Jiang L (2021). tsRNA-5001a promotes proliferation of lung adenocarcinoma cells and is associated with postoperative recurrence in lung adenocarcinoma patients. Transl Lung Cancer Res.

[92] Han L, Lai H, Yang Y, Hu J, Li Z, Ma B, Xu W, Liu W, Wei W, Li D (2021). A 5'-tRNA halve, tiRNA-Gly promotes cell proliferation and migration via binding to RBM17 and inducing alternative splicing in papillary thyroid cancer. J Exp Clin Cancer Res.

[93] Lone SN, Nisar S, Masoodi T, Singh M, Rizwan A, Hashem S, El-Rifai W, Bedognetti D, Batra SK, Haris M (2022). Liquid biopsy: a step closer to transform diagnosis, prognosis and future of cancer treatments. Mol Cancer.

[94] Li J, Zhu L, Cheng J, Peng Y (2021). Transfer RNA-derived small RNA: a rising star in oncology. Semin Cancer Biol.

[95] Zhu L, Li T, Shen Y, Yu X, Xiao B, Guo J (2019). Using tRNA halves as novel biomarkers for the diagnosis of gastric cancer. Cancer Biomark.

[96] Wang J, Ma G, Ge H, Han X, Mao X, Wang X, Veeramootoo JS, Xia T, Liu X, Wang S (2021). Circulating tRNA-derived small RNAs (tsRNAs) signature for the diagnosis and prognosis of breast cancer. NPJ Breast Cancer.

[97] Wang J, Liu X, Cui W, Xie Q, Peng W, Zhang H, Gao Y, Zhang C, Duan C (2022). Plasma tRNA-derived small RNAs signature as a predictive and prognostic biomarker in lung adenocarcinoma. Cancer Cell Int.

[98] Wu Y, Yang X, Jiang G, Zhang H, Ge L, Chen F, Li J, Liu H, Wang H (2021). 5'-tRF-GlyGCC: a tRNA-derived small RNA as a novel biomarker for colorectal cancer diagnosis. Genome Med.

[99] Feng W, Li Y, Chu J, Li J, Zhang Y, Ding X, Fu Z, Li W, Huang X, Yin Y (2018). Identification of tRNA-derived small noncoding RNAs as potential biomarkers for prediction of recurrence in triple-negative breast cancer. Cancer Med.

[100] Sun C, Yang F, Zhang Y, Chu J, Wang J, Wang Y, Zhang Y, Li J, Li Y, Fan R (2018). tRNA-derived fragments as novel predictive biomarkers for trastuzumab-resistant breast cancer. Cell Physiol Biochem.

[101] Huang Y, Ge H, Zheng M, Cui Y, Fu Z, Wu X, Xia Y, Chen L, Wang Z, Wang S, Xie H (2020). Serum tRNA-derived fragments (tRFs) as potential candidates for diagnosis of nontriple negative breast cancer. J Cell Physiol.

[102] Mo D, He F, Zheng J, Chen H, Tang L, Yan F (2021). tRNA-derived fragment tRF-17-79MP9PP attenuates cell invasion and migration via THBS1/TGF-β1/Smad3 axis in breast cancer. Front Oncol.

[103] Zhan S, Yang P, Zhou S, Xu Y, Xu R, Liang G, Zhang C, Chen X, Yang L, Jin F, Wang Y (2022). Serum mitochondrial tsRNA serves as a novel biomarker for hepatocarcinoma diagnosis. Front Med.

[104] Lin C, Zheng L, Huang R, Yang G, Chen J, Li H (2020). tRFs as potential exosome tRNA-derived fragment biomarkers for gastric carcinoma. Clin Lab.

[105] Zhu L, Li J, Gong Y, Wu Q, Tan S, Sun D, Xu X, Zuo Y, Zhao Y, Wei YQ (2019). Exosomal tRNA-derived small RNA as a promising biomarker for cancer diagnosis. Mol Cancer.

[106] Li K, Lin Y, Luo Y, Xiong X, Wang L, Durante K, Li J, Zhou F, Guo Y, Chen S (2022). A signature of saliva-derived exosomal small RNAs as predicting biomarker for esophageal carcinoma: a multicenter prospective study. Mol Cancer.

[107] Wang J, Ma G, Li M, Han X, Xu J, Liang M, Mao X, Chen X, Xia T, Liu X, Wang S (2020). Plasma tRNA fragments derived from 5′ ends as novel diagnostic biomarkers for early-stage breast cancer. Mol Ther Nucleic Acids.

[108] Xue M, Shi M, Xie J, Zhang J, Jiang L, Deng X, Peng C, Shen B, Xu H, Chen H (2021). Serum tRNA-derived small RNAs as potential novel diagnostic biomarkers for pancreatic ductal adenocarcinoma. Am J Cancer Res.

[109] Gupta N, Singh A, Zahra S, Kumar S (2018). PtRFdb: a database for plant transfer RNA-derived fragments. Database.

[110] Thompson A, Zielezinski A, Plewka P, Szymanski M, Nuc P, Szweykowska-Kulinska Z, Jarmolowski A, Karlowski WM (2018). tRex: a web portal for exploration of tRNA-derived fragments in *Arabidopsis*
*thaliana*. Plant Cell Physiol.

[111] Pliatsika V, Loher P, Magee R, Telonis AG, Londin E, Shigematsu M, Kirino Y, Rigoutsos I (2018). MINTbase v2.0: a comprehensive database for tRNA-derived fragments that includes nuclear and mitochondrial fragments from all The Cancer Genome Atlas projects. Nucleic Acids Res.

[112] Yao D, Sun X, Zhou L, Amanullah M, Pan X, Liu Y, Liang M, Liu P, Lu Y (2020). OncotRF: an online resource for exploration of tRNA-derived fragments in human cancers. RNA Biol.

[113] Zhou Y, Peng H, Cui Q, Zhou Y (2021). tRFTar: prediction of tRF-target gene interactions via systemic re-analysis of Argonaute CLIP-seq datasets. Methods.

[114] Xiao Q, Gao P, Huang X, Chen X, Chen Q, Lv X, Fu Y, Song Y, Wang Z (2021). tRFTars: predicting the targets of tRNA-derived fragments. J Transl Med.

[115] Wang JH, Chen WX, Mei SQ, Yang YD, Yang JH, Qu LH, Zheng LL (2022). tsRFun: a comprehensive platform for decoding human tsRNA expression, functions and prognostic value by high-throughput small RNA-Seq and CLIP-Seq data. Nucleic Acids Res.

[116] Li N, Shan N, Lu L, Wang Z (2021). tRFtarget: a database for transfer RNA-derived fragment targets. Nucleic Acids Res.

[117] Zuo Y, Zhu L, Guo Z, Liu W, Zhang J, Zeng Z, Wu Q, Cheng J, Fu X, Jin Y (2021). tsRBase: a comprehensive database for expression and function of tsRNAs in multiple species. Nucleic Acids Res.

